# Question-writing as a learning tool for students – outcomes from curricular exams

**DOI:** 10.1186/1472-6920-13-89

**Published:** 2013-06-21

**Authors:** Alexander Jobs, Christoph Twesten, Anna Göbel, Hendrik Bonnemeier, Hendrik Lehnert, Gunther Weitz

**Affiliations:** 1 Department of Internal Medicine II, University Hospital of Schleswig-Holstein, Campus Lübeck, Ratzeburger Allee 160, 23538, Lübeck, Germany; 2 Department of Internal Medicine I, University Hospital of Schleswig-Holstein, Campus Lübeck, Ratzeburger Allee 160, 23538, Lübeck, Germany; 3 Medical Section, University of Lübeck, Hs. 23A, Ratzeburger Allee 160, 23538, Lübeck, Germany; 4 Department of Internal Medicine III, University Hospital of Schleswig-Holstein, Campus Kiel, Arnold-Heller-Straße 3, Kiel, 24105, Germany

**Keywords:** Question-writing, Multiple choice questions, Curricular exams, Learning behavior, Internal medicine

## Abstract

**Background:**

Writing exam questions can be a valuable learning tool. We asked students to construct multiple choice questions for curricular exams in Internal Medicine. The questions for the particular exams were chosen from a pool of at least 300 student-written questions. The uncorrected pool was accessible to all students. We studied the influence of this approach on the students’ learning habits and their test results. We hypothesized that creating a pool of their own questions for the exams could encourage students to discuss the learning material.

**Methods:**

All students had to pass 4 exams in 7 fields of Internal Medicine. Three exams were comprised of 20 questions, and we applied the new method in one of these exams. The fourth exam was comprised of 30 questions, 15 of which were chosen from a students’ pool. After all exams had been completed we asked the students to fill in a web-based questionnaire on their learning habits and their views on the new approach. The test-results were compared to the results of the lecturers’ questions that defined high and low performing students.

**Results:**

A total of 102 students completed all four exams in a row, 68 of whom filled in the questionnaire. Low performing students achieved significantly better results in the students’ questions. There was no difference in the number of constructed questions between both groups of students. The new method did not promote group work significantly. However, high performing students stated a stronger wish to be rewarded by good performance.

**Conclusions:**

Creating a curricular exam by choosing questions from a pool constructed by students did not influence the learning habits significantly and favored low performing students. Since the high performing students sought to be rewarded for their efforts, we do not consider the approach applied in our study to be appropriate.

## Background

Most lecturers have probably experienced the difficulties of constructing valid questions for written exams. Apart from the different possible question formats [1] the content of the questions ought to be correct and able to withstand scientific challenge. In some cases a thorough review of literature is necessary in order to exclude misconceptions. This process can be used as a learning tool for students. It has been reported that constructing questions enhances the recall for a studied text [2]. It may also foster a more active and self-determined way of learning [3] which is more likely to promote a deeper understanding of the subject [4]. Moreover, applying self-written questions in exams may create a constructive learning climate and reduce anxiety [5].

The construction of questions has occasionally been used as a tool in medical education: In a contest during a physiology class, 37 out of 100 students submitted a total of 912 questions [6]. These questions were graded and discussed in the class. The authors state that question writing motivated students to study and that lively discussions occurred. In another study the construction of multiple choice questions (MCQs) was used as a stimulus for learning in clinical surgery [7]. The questions submitted were of high quality; however, the studied group of students did not achieve better results in the exams than a control group. The method was unpopular at first but was rated more favourable after the exercise.

More recently, Yu and Liu studied the effects of question-posing as compared to question-answering during weekly post-lecture sessions in a cohort of 69 civil engineering sophomores [8]. While there was no difference in academic achievement the question-posing group had significantly higher abilities in cognitive and metacognitive learning strategies after the intervention. Denny and co-workers developed a web-based system in which students create MCQs and answer those created by their peers (http://peerwise.cs.auckland.ac.nz). Students using this system have been shown to produce good quality MCQs and use higher order thinking skills while taking an active role in their learning [9].

Applying self-written questions in curricular medical exams could have substantial impact on the students’ learning behaviour. In a recent study, 25% of the MCQs in an end-of-year formal examination were derived from a bank of student-generated questions [10]. Many students, however, chose to memorise the question bank as a “high‒yield” strategy for mark inflation, favouring surface rather than deep learning. To date, the literature on the impact of student-generated questions in curricular exams is scarce. In 2010, the student council of our university asked the lecturers of Internal Medicine whether students could submit questions for the exams in this field. The student council would collect the questions and provide a pool of at least 300 appropriate questions per exam to the lecturers. The lecturers would then select the exam questions from the pool. The eventual motive was to achieve a more problem-based approach to learning thereby promoting team work. After a discussion, the advisory board agreed to apply this method in two of four exams. Hence, we had a unique opportunity to study the effects of this procedure in comparison to questions constructed by the lecturers. The main goal of the study was to evaluate the effect on the students’ learning habits in comparison to the conventional approach. We were also interested in the impact of the method on the grade distribution.

## Methods

In our faculty, Internal Medicine is taught in the fourth year of medical studies. The subjects in this field are allocated into four sections and there is a written exam for each section: Endocrinology and Nephrology (EN), Gastroenterology, Hematology, and Oncology (GHO), Respiratory Medicine and Cardiology (RMC), and Rheumatology (Rh). The students can choose to be tested in the particular subjects in the fourth year or later. During the studied period (2011), the exams consisted of 20 (EN, RMC, and Rh) and 30 (GHO) MCQs. All the questions in EN and RMC, and 15 of the questions in GHO were constructed by the lecturers themselves and underwent a review process by the teaching advisory board of Internal Medicine. All 20 questions in Rh and 15 of the questions in GHO were derived from a pool of at least 300 questions which had been submitted by students three weeks before the exam.

All students had been instructed in writing MCQs using an approved manual, which was made accessible to the students by the students’ council. The advice given by the manual referred to the question type (type-A questions with five choices and one correct answer), the structure (how to weigh stem and options, how to avoid cues), the content (reference to learning objective, relevance), and the hierarchy of knowledge (preference of higher-order skills). Questions were only eligible for the pool if they were of acceptable quality. The selection from this pool was made by the lecturers and the selected questions underwent the same review process through the advisory board as the questions constructed by the lecturers themselves. All the questions were assessed by their correctness (with regard to form, language and content) and their relevance (according to the stated learning objectives). The questions for the exam were chosen from the best proposals in order to cover a broad section of the field and to exclude cues by question interference. The reviewers felt that the quality of the selected questions was not significantly different between the students' and the lecturers' proposals. However, they did not rate the questions numerically. To avoid the possibility that students could guide the selection process by intentionally submitting incorrect questions (which then would be excluded), both lecturers and the advisory board were allowed to make minor corrections. The pool of the submitted (possibly incorrect) questions was accessible to all students.

After all exams had been completed, the tested students were asked via e-mail to fill in a web-based questionnaire. In the questionnaire the students were asked to state the number of questions they had constructed and the hours they had spent studying for the particular exam. They also reported the amount of time they spent using variable learning methods: studying lecture slides or textbooks, working in a group, creating questions, studying the pool of the students’ questions and others (studying clinical guidelines, other pools of questions, web-based resources or miscellaneous). Additionally, they could rate their attitude towards the exam in a 5-point-true-false-scale. In order to combine the students’ results of the exams with the answers given in the questionnaire, their matriculation number was used. All students who filled in the questionnaire gave informed consent for processing their data. The pooled data were further processed without identification of the students. The study was approved by means of the ethics committee of the University of Lübeck. The work was carried out in accordance with the Declaration of Helsinki, and the anonymity of all participants was guaranteed.

We assessed the results of the students who had taken all four exams (n = 102). For this purpose, we pooled the 55 lecturer-written questions (EN, GHO, and RMC) and the 35 student-written questions (GHO and Rh). We differentiated high and low performing students based on the lecturers’ questions, dividing the students into tertiles: Students in the highest tertile were defined as high performing, students in the lowest tertile as low performing (Table 1). The GHO exam was comprised of both lecturers’ and students’ questions. We therefore evaluated this exam separately and compared the results to the overall results.

**Table 1:** Performance of the three groups of students divided into tertiles by the results of the lecturers’ questions

	**Relative performance [%] of students who took all four exams (n = 102)**			**Relative performance [%] of students who took all four exams and filled in the questionnaire (n = 68)**		
	**Lecturers’ questions**	**Students’ questions**	**n**	**Lecturers’ questions**	**Students’ questions**	**n**
	**(mean ± SD)**	**(mean ± SD)**		**(mean ± SD)**	**(mean ± SD)**	
high performer	87.8 ± 3.4	93.9 ± 3.3	32	86.8 ± 3.7	92.9 ± 4.2	21
intermediate performer	78.5 ± 2.1	90.9 ± 4.5	28	77.9 ± 1.5	91.0 ± 3.1	21
low performer	69.4 ± 5.3	88.5 ± 5.1	42	68.9 ± 6.1	87.9 ± 6.5	26

**Table 2:** Results of the questionnaire

	**High performer (n=21)**	**Low performer (n=26)**	**P-value**
	**median [IQR]**	**median [IQR]**	
The grade in internal medicine is important to me.	1 [1-2]	2 [1-3]	<.01
I consider the grade in internal medicine to be important for future job applications.	2 [1-3]	3 [1-3]	<.05
I find it important that the exams in internal medicine differentiate between high and low performing students.	3 [2-3]	4 [4-4]	<.001
I consider a good grade be a reward for my learning efforts.	2 [1-2]	2 [2-2]	n.s.
I have learned more in the system with the students’ questions than in the system with the lecturers’ questions.	3 [3-4]	3 [2-4]	n.s.
Number of constructed questions	6 [3-10]	6 [0-9]	n.s.

There was a 5-point-true-false-scale for the statements: 1=completely true, 2 = rather true, 3 = neutral, 4 = rather false, 5 = completely false. Data are presented as mean and interquartile range. The P-values indicate the results of a Mann-Whitney U test comparing the groups.

### Statistics

Data management, statistical analysis, and graphical presentation were performed using the *R* software environment [11]. The results of the exams are given as percentage of the achievable correct answers (relative performance; mean ± SD). The Welch test was applied for the comparison of high and low performing students. The results of the ordinal scaled questionnaire and the numbers of constructed questions per student are presented as median [interquartile range] and compared by the Mann–Whitney U test. The total time spent in preparation for the exams was compared using the analysis of variance (ANOVA) followed by pairwise testing with adjustment for multiple testing according to Bonferroni. The particular amount of time in hours spent with different learning methods was compared between high and low performing students using the Welch test. A *P*-value  < .05 was considered significant.

## Results

**Figure 1:**
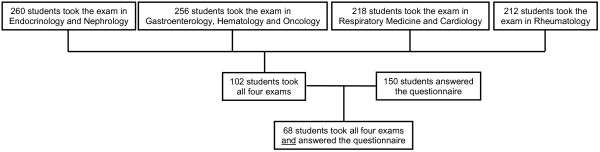
Number of students taking the exams and filling in the questionnaire.

**Figure 2:**
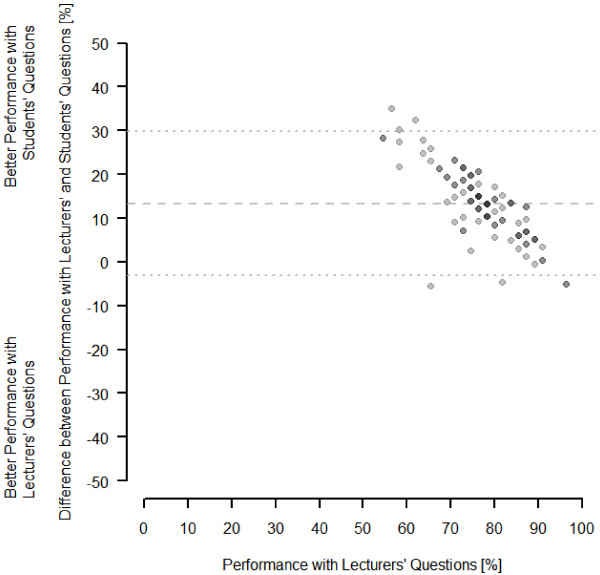
**Bland-Altman-plot of the performance of the students who took all four exams (n = 102) differentiating by the type of questions.** The gray saturation indicates whether data points superpose a xy-pair (up to 5 students per point).

The number of students who took the exams and filled in the questionnaire is given in Figure 1. A total of 102 students took all four exams, two thirds (66.7%) of whom filled in the questionnaire. As shown in Table 1, the characteristics of the latter were highly consistent with the whole group. The low performing students achieved significantly better results answering the students’ questions while the high performing students did not have a measureable advantage under this system (+19.0% versus +6.1%, respectively; Table 1; Figure 2). In this respect, the differences between these two groups were highly significant (*P *< .001). The groups also significantly differed in their attitude towards the exams (Table 2): The grade in Internal Medicine appeared more important to the high performing group and this group felt stronger that the grade in Internal Medicine would be important for future job applications. Moreover, there was a stronger wish in this group that the exams could differentiate between high and low performing students. There was no difference in the number of questions submitted by the two groups (Tables 2 and 3).

**Table 3:** Distribution of students who constructed up to three questions versus students who constructed six or more questions to the high and low performing group, respectively

	**High performer**	**Low performer**	**Total**
≥6 constructed questions	14	16	30
≤3 constructed questions	6	9	15

The evaluation of learning methods is given in Table 4. The most time was spent in private sessions studying lecture slides and textbooks; little time was spent studying in peer groups. There was no difference in this trend with regard to the system applied. Moreover, the students spent relatively little time in constructing the questions. During the preparation for the GHO-exam with both students’ and lecturers’ questions, the students spent relatively little time on studying the pool of questions. However, in the Rh-exam consisting of students’ questions only, studying the pool of questions was a highly appreciated learning method and it was even the preferred method in the group of low performing students.

**Table 4:** Hours spent preparing for the four exams according to different learning methods as recalled in the questionnaire

	**Time spent preparing for an exam in hours**							
	**EN-exam**		**GHO-exam**		**RMC-exam**		**Rh-exam**	
	**High performer**	**Low performer**	**High performer**	**Low performer**	**High performer**	**Low performer**	**High performer**	**Low performer**
Lecture	17 ±3	12 ±2	14 ±2	12 ±2	16 ±3	12 ±2	5 ±1	3 ±1
Textbook	20 ±4	18 ±3	16 ±2	15 ±2	15 ±2	15 ±2	6 ±1	7 ±1
Peer group	3 ±1	3 ±1	3 ±1	3 ±1	3 ±1	2 ±1	1 ±0	2 ±1
Construction of questions	n.a.	n.a.	2 ±0	0 ±0 *	n.a.	n.a.	1 ±0	1 ±1
pool of questions	n.a.	n.a.	5 ±1	4 ±1	n.a.	n.a.	6 ±1	7 ±1
other	9 ±1	9 ±1	5 ±1	5 ±1	8 ±1	10 ±2	2 ±1	2 ±1
∑	49 ±5	42 ±4	45 ±4	41 ±4	42 ±4	39 ±4	20 ±2	22 ±2

Data are presented as mean ±SEM; n.a. = not applicable. *P<.05 comparing high and low performers.

**Figure 3:**
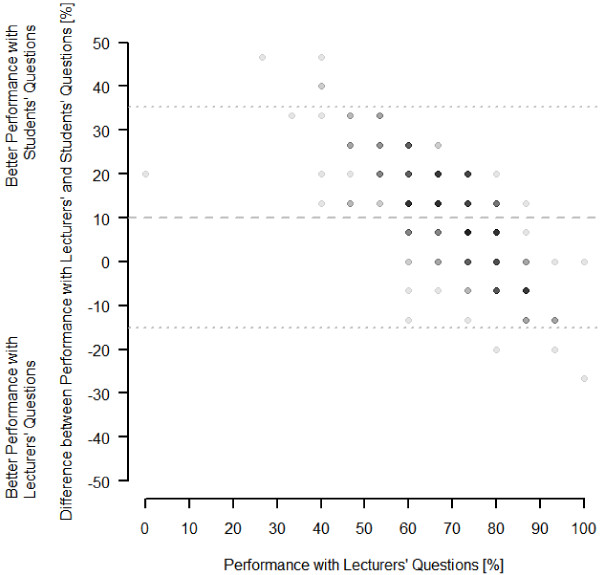
**Bland-Altman-plot of the performance of the students who took the GHO-exam (n = 256) differentiating by the type of questions.** The gray saturation indicates whether data points superpose a xy-pair (up to 19 students per point).

The separate analysis of the results of the GHO-exam (n = 254) revealed comparable results to the analysis of all exams (Figure 3): The high performing students achieved only 4.1% better results in the 15 questions by the students as compared to the 15 lecturers questions. The group of low performing students achieved 13.3% better results. The difference between these two rates was significant (*P *< .05).

The calculation of the difficulty and discrimination index of students' (n = 35) and lecturers' questions (n = 55) revealed that the lecturers' questions were more difficult (0.78 versus 0.90, respectively; P < .001) and discriminated better (discrimination index 0.18 versus 0.12, respectively; P < .05) than the students' questions.

## Discussion

In this study, we report on the effects of students writing questions for their own exams. Writing questions is challenging and might encourage a more intense approach to learning because the writer has to scrutinize and question his own views. All students’ questions were accessible to the learners, however, only in the original, uncorrected students’ version. It was therefore an additional idea to create a discussion among the students about the correctness of the questions, hence promoting team work.

The main result of the study is that our approach did not measurably influence the students’ learning habits in the way we intended. Little time was spent constructing the questions and studying in a group was a consistently unpopular learning method. The time studying the pool of questions by far exceeded the time constructing questions. In preparation for the exam with both types of questions (GHO) the students did not schedule their time differently compared to the exams with lecturers' questions. This suggests that when it comes to answering unknown questions the students would rather rely on their conventional approach to learning than on getting involved in writing and discussing their own questions. The overall time studying for the exam with students' questions only (Rh) was significantly lower than for the other exams (*P *< .001) and the students spent a great deal of their time memorising the question pool. This may indicate that medical students indeed are strategic learners trying to be time efficient in their preparation for the exams [12] rather than seeking deeper understanding. However, there may be alternative explanations for the reduced time the students spent learning for the latter exam: Firstly, only one subject in the field of Internal Medicine was tested and, secondly, the exam took place at the end of the semester competing with other exams in the same group of students. Nevertheless, the studied method did not promote learning with a peer group as it intended.

With regards to the test results, low performing students achieved better results while the high performing students did not have any advantage. This may be interpreted as a desirable result because the weaker students were supported. However, due to the fact that the same results occurred in the mixed exam with both students’ and lecturers’ questions (GHO) it is more likely that the weaker students achieved better by learning the pool of questions by heart rather than by understanding the material on a deeper level. The high performing students also spent a lot of time studying the pool, however, they did not achieve a significantly better score. This may be due to a ceiling effect implying that they had already obtained their best possible score.

Exams can serve several purposes: they may be used to assign a grade, and/or to provide feedback to the students and information to the teacher about what the students did not understand [13]. From this point of view the review of the students’ questions allowed the lecturers a good overview of the students’ deficits, and, moreover, of what they perceived to be important. This knowledge can be used to realign the lectures. On the other hand, the self-written exam was neither able to assign a correct grade nor give informative feedback to the students because the greater part of the corrective was in the students’ hands in advance of the exam.

Superordinate goals of an assessment in medical education are to optimize the capabilities of all learners by providing motivation and direction for future learning, to protect the public by identifying incompetent candidates, and to provide a basis for choosing applicants for advanced training [14]. Hence, the most appropriate concept of fairness of an exam is probably whether it is testing what makes the better professional [13]. The grades of the self-written exams did not reflect the appreciation of the lecturers. In our setting all lecturers were experienced physicians. As we have previously shown, physicians have a different view of what is important in medical education than students [15]. From a professional point of view, physicians may be in a better position to oversee the requirements for a medical graduate than the medical students themselves. Given that their questions reflected these requirements, their questions would better fulfil the criteria of fairness than the students’ questions. The disadvantage for the high performing students was also more obvious due to the fact that there was a significantly greater wish for discrimination between high and low achievement as well as a greater sense of the importance of the grade among these higher performing students.

There are, however, several limitations with this study. The number of students studied was limited. We only included the students who chose to take all four exams in a row. Less than half of the cohort fulfilled this criterion, two thirds of whom filled in the questionnaire. However, we believe that as there was no detectable positive effect on learning habits in a group of this size, the overall benefit of the method is likely to be too small to justify the effort of curricular implementation. Secondly, while question writing seems to be a valuable learning tool, we can only speak for the studied method. The procedure was decided on before the study; hence, the study had an observational character. A different approach might have led to a different outcome. A web-based platform to create and discuss the questions as previously described [9] could have promoted a broader interest in the process of scrutinising the correctness of the questions. Also, a modification in handling the pool (e.g. number of questions, introducing a rating, limiting to reviewed questions) could have changed the results. Letting the students write the questions for their own exam might therefore indeed be a valuable learning tool [8]. However, we were not able to find a positive influence of our method on the learning behaviour and abandoned it in the curriculum.

## Conclusions

Letting students write their own exam in the described manner does not appear to have a beneficial influence on learning habits. Low performing students achieve better by learning the questions by heart. Since the high performing students in our study wished their achievements to be acknowledged we considered the method unfair.

## Abbreviations

MCQ: Multiple choice question; EN: Endocrinology and nephrology; GHO: Gastroenterology hematology, and oncology; RMC: Respiratory medicine and cardiology; Rh: Rheumatology.

## Competing interests

The authors declare that they have no competing interests.

## Authors’ contributions

AJ had the idea of publishing the data, performed the questionnaire together with CT, did all the statistics, created the figures, and was, together with the other authors, involved in the discussions and in writing the article. CT performed the questionnaire, collected the data, and was involved in the discussions on the results and in writing the paper. AG, HB and HL gave important advice for the questionnaire, were involved in the discussions on the results and in writing the paper. GW coordinated all the actions, moderated the discussions, wrote the first version of the article, and submitted the final version. All authors approved this version.

## Pre-publication history

The pre-publication history for this paper can be accessed here:

http://www.biomedcentral.com/1472-6920/13/89/prepub
